# Phenotypic and genetic spectrum of isolated macrodactyly: somatic mosaicism of *PIK3CA* and *AKT1* oncogenic variants

**DOI:** 10.1186/s13023-020-01572-9

**Published:** 2020-10-14

**Authors:** Wen Tian, Yingzhao Huang, Liying Sun, Yang Guo, Sen Zhao, Mao Lin, Xiying Dong, Wenyao Zhong, Yuehan Yin, Zefu Chen, Nan Zhang, Yuanqiang Zhang, Lianlei Wang, Jiachen Lin, Zihui Yan, Xinzhuang Yang, Junhui Zhao, Guixing Qiu, Jianguo Zhang, Zhihong Wu, Nan Wu, (Deciphering Disorders Involving Scoliosis, COmorbidities) study group

**Affiliations:** 1 Department of Hand Surgery, Beijing Jishuitan Hospital, Beijing, 100035 China; 2 Department of Orthopedic Surgery, Peking Union Medical College Hospital, Peking Union Medical College, Chinese Academy of Medical Sciences, No. 1 Shuaifuyuan, Beijing, 100730 China; 3 Beijing Key Laboratory for Genetic Research of Skeletal Deformity, Beijing, 100730 China; 4 Medical Research Center of Orthopedics, Chinese Academy of Medical Sciences, Beijing, 100730 China; 5 Department of Central Laboratory, Peking Union Medical College Hospital, Peking Union Medical College, Chinese Academy of Medical Sciences, No. 1 Shuaifuyuan, Beijing, 100730 China

**Keywords:** Macrodactyly, Phosphatidylinositol 3-kinase catalytic subunit alpha (*PIK3CA*), *AKT1* serine/threonine kinase 1 (*AKT1*), Somatic mosaicism, Proteus syndrome

## Abstract

**Background:**

Isolated macrodactyly is a severe congenital hand anomaly with functional and physiological impact. Known causative genes include *PIK3CA*, *AKT1* and PTEN. The aim of this study is to gain insights into the genetics basis of isolated macrodactyly.

**Results:**

We enrolled 24 patients with isolated macrodactyly. Four of them were diagnosed with Proteus syndrome based on skin presentations characteristic to this disease. Targeted next-generation sequencing was performed using patients’ blood and affected tissues. Overall, 20 patients carry mosaic *PIK3CA* pathogenic variants, i.e. p.His1047Arg (N = 7), p.Glu542Lys (N = 6), p.Glu545Lys (N = 2), p.His1047Leu (N = 2), p.Glu453Lys (N = 1), p.Gln546Lys (N = 1) and p.His1047Tyr (N = 1). Four patients who met the diagnostic criteria of Proteus syndrome carry mosaic *AKT1* p.Glu17Lys variant. Variant allele frequencies of these mosaic variants obtained through next-generation sequencing range from 10 to 33%. In genotype–phenotype correlation analysis of patients with *PIK3CA* variant, we found that patients with the macrodactyly of the lower limbs tend to carry *PIK3CA* variants located in the helical domain (*P* = 0.005).

**Conclusions:**

Mosaic *PIK3CA* and *AKT1* variants can be found in all of our samples with isolated macrodactyly. Insights into phenotypic and genetic spectrum of isolated macrodactyly may be helpful in perusing a more precise and effective management of isolated macrodactyly.

## Introduction

Macrodactyly is a rare congenital anomaly characterized by the overgrowth of digits on one or multiple limbs, which can present as either isolated or syndromic (in conjunction with other congenital defects) macrodactyly. It occurs in approximately 1/50,000–1/100,000 live births, and varies according to regional and ethnical demographics [1, 2]. The associated dysmorphic appearance and cultural stigma have the potential to lead to psychological and social problems that can persist through to adulthood. Due to the highly variable phenotypic manifestation and low frequency of occurrence, there is no standardized protocol for the treatment of macrodactyly, presenting a unique challenge for surgeons faced with correcting abnormalities [3]. Currently, surgical procedures such as soft tissue debulking, physeal arrest and amputation are used to produce acceptable functional and cosmetic outcomes, but novel therapeutic strategies are still needed [5].

Somatic mosaicism of phosphatidylinositol 3-kinase catalytic subunit alpha (*PIK3CA*) mutations has been identified as the cause of multiple overgrowth disorders, including isolated macrodactyly, congenital lipomatous overgrowth, vascular malformations, epidermal nevi and skeletal/scoliosis/spinal abnormalities (CLOVES syndrome) [4], Kippel–Trenaunay syndrome (KTS) [5], megalencephaly-capillary malformation (MCAP) [6], dysplastic megalencephaly (DMEG) [7], and capillary malformation of the lower lip, lymphatic malformation of the face and neck, and asymmetry and partial/generalized overgrowth (CLAPO syndrome) [8]. Despite their clinical differences, tissue enlargement represents a distinct feature common to all of these disorders. *PIK3CA* is one of the most frequently mutated genes in human tumors [9], encoding the alpha catalytic subunit of phosphatidylinositol-4,5-bisphosphate 3-kinase, a member of the phosphatidylinositol 3-kinase (PI3K) enzyme family [9]. Signaling through PI3K-AKT-mTOR mediates cell proliferation, survival and metabolism through enhancement of lipid kinase activity, and constitutive activation of this pathway through mutations in *PI3K3CA* is well established in tumorigenesis [10].

While the correlation between *PIK3CA* and *AKT1* mutations in macrodactyly is well characterized [11], the mutational architecture of somatic mosaicism and its effect on phenotypic presentation in isolated macrodactyly is still not well understood, particularly due to the challenges of identifying of low-level mosaicism in affected tissue. Currently, research employing next generation sequencing (NGS)-based methods to explore the genetic basis of isolated macrodactyly hold much promise. In this study, we utilize an NGS-based strategy to identifiy mosaic *PIK3CA* and *AKT1* variants in a cohort of 24 isolated macrodactyly patients. Furthermore, we assess the genotype–phenotype correlation caused by distinct *PIK3CA* variants in isolated macrodactyly.

## Results

### Clinical characteristics of the subjects

**Fig. 1:**
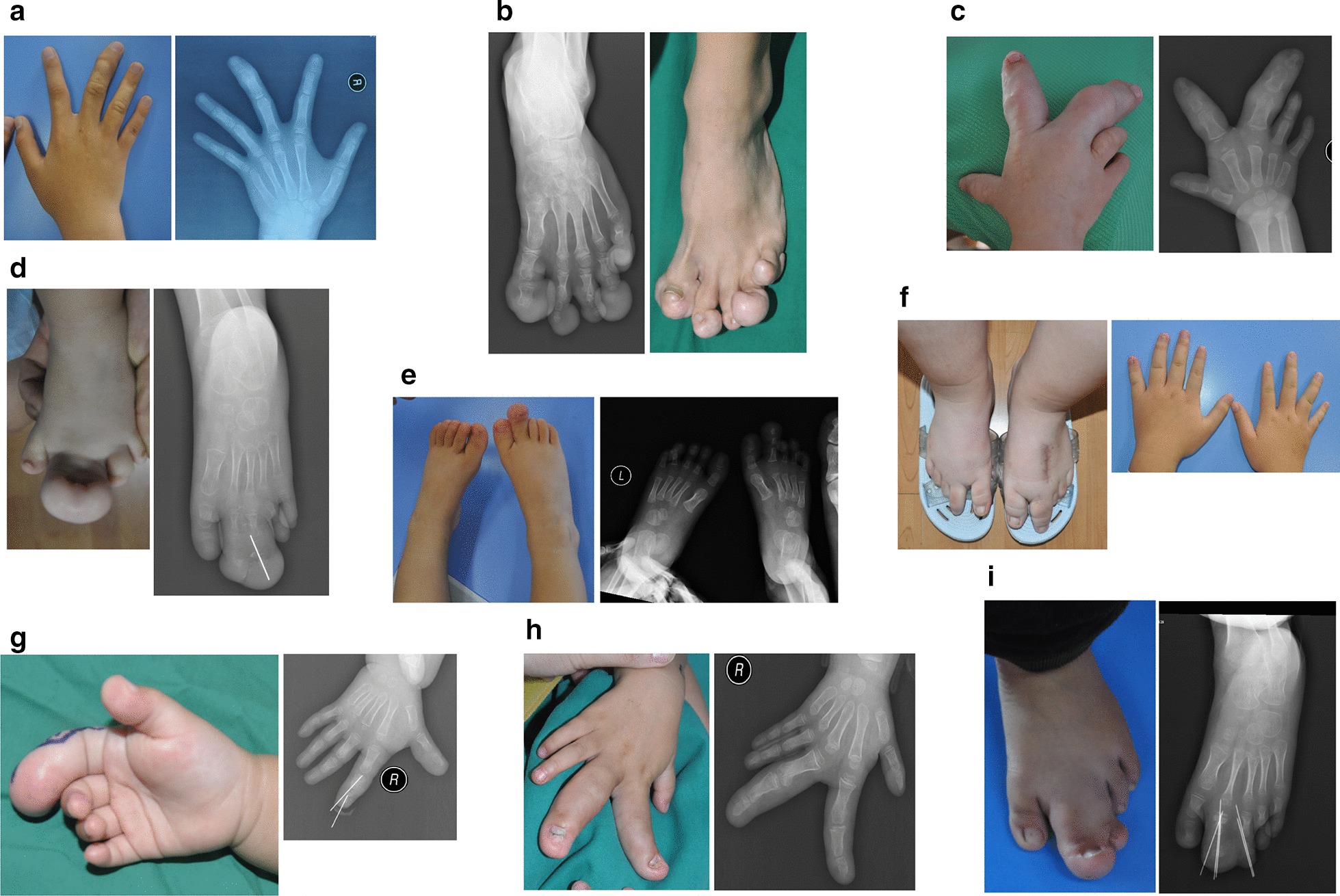
Representative clinical photographs of nine macrodactyly patients. **a** Patient No. 1. **b** Patient No. 2. **c** Patient No. 3. **d** Patient No. 18. **e** Patient No. 6. **f** Patient No. 15. **g** Patient No. 19. **h** Patient No. 16. **i** Patient No. 21

Clinical and molecular characteristics of these 24 subjects are described in Table 1. Representative photographs of nine isolated macrodactyly patients are shown in Fig. 1. 22 of 24 patients displayed asymmetric and disproportionate overgrowth in their hands or feet at birth, while the remaining 2 patients had later onset symptoms between six and twelve months after birth. With the exception of one 34 years old, the majority of patients were under 15 years old, with a median age of 5. A slight male predominance was observed (16 males vs. 8 females, binomial *P* = 0.152) in our cohort. 14 patients presented exclusive involvement of the lower extremities, while nine had macrodactyly exclusively in the upper extremities (binomial *P* = 0.405). Only one patient had overgrowth in both the upper and the lower extremities. Twenty of the 24 patients had unilateral involvement, with 12 patients presenting on the right side and eight patients presenting on the left side of the body (binomial *P* = 0.503). The number of affected digits ranged from one to seven, with an average of 2.7 digits affected. The second digit was the most frequently affected digit (N = 22), followed by the third (N = 17). Over half of our patients (14/24) had two affected digits, and the combination of second- and third-digit overgrowth (N = 10) was more frequently observed than the combination of first- and second-digit enlargement (N = 4; binomial *P* = 0.180). In addition, no other combinations were observed in patients with two affected digits. 5 of the 24 patients had syndactyly, a condition where two or more digits are fused together, all of which presented as syndactyly of 2–3 toes.

**Table 1:** Summary of the clinical and molecular findings of studied subjects

Patient nos.	Gender	Age	Syndactyly	Skin findings	Affected digits	Gene	Variant	VAF (%)
1	M	11	N	CCTN	R-Hand: 2,3	*AKT1*	c.49G > A (p.Glu17Lys)	22.03
2	M	11	N	CCTN	L-Foot: 1,2,3,4,5	*AKT1*	c.49G > A (p.Glu17Lys)	11.16
3	M	2	N	CCTN	R-Hand: 2,3	*AKT1*	c.49G > A (p.Glu17Lys)	9.93
4	F	10	N	CCTN	L-Hand: 3,4,5	*AKT1*	c.49G > A (p.Glu17Lys)	20.57
5	F	6	N	N	L-Foot: 2,3; R-Foot:1.2; R-Hand:2,3	*PIK3CA*	c.1357G > A (p.Glu453Lys)	11.10
6	M	3	N	N	R-Foot: 2	*PIK3CA*	c.1624G > A (p.Glu542Lys)	24.48
7	M	4	Y	N	L-Foot: 2,3	*PIK3CA*	c.1624G > A (p.Glu542Lys)	17.15
8	M	5	Y	N	L-Foot: 1,2,3,4	*PIK3CA*	c.1624G > A (p.Glu542Lys)	20.95
9	M	4	N	N	L-Foot: 2,3	*PIK3CA*	c.1624G > A (p.Glu542Lys)	17.10
10	F	4	N	N	R-Foot: 2,3	*PIK3CA*	c.1624G > A (p.Glu542Lys)	27.58
11	M	3	N	N	R-Foot: 1,2	*PIK3CA*	c.1624G > A (p.Glu542Lys)	17.79
12	M	2	N	N	L-Foot: 1,2,3	*PIK3CA*	c.1633G > A (p.Glu545Lys)	19.11
13	M	2	Y	N	R-Foot: 2,3	*PIK3CA*	c.1633G > A (p.Glu545Lys)	27.31
14	M	11	N	N	L-Foot: 1,2,3; R-Foot:1,2,3,4	*PIK3CA*	c.1636C > A (p.Gln546Lys)	24.50
15	F	13	N	N	B-Feet: 2	*PIK3CA*	c.3139C > T (p.His1047Tyr)	18.94
16	M	1	N	N	R-Hand: 2,3	*PIK3CA*	c.3140A > G (p.His1047Arg)	25.63
17	M	2	Y	N	R-Foot: 1,2	*PIK3CA*	c.3140A > G (p.His1047Arg)	23.29
18	F	5	Y	N	L-Foot: 2,3	*PIK3CA*	c.3140A > G (p.His1047Arg)	21.45
19	M	2	N	N	R-Hand: 1,2	*PIK3CA*	c.3140A > G (p.His1047Arg	25.57
20	F	10	N	N	R-Hand: 1,2,3,4,5	*PIK3CA*	c.3140A > G (p.His1047Arg)	10.36
21	M	2	N	N	L-Foot: 2,3	*PIK3CA*	c.3140A > G (p.His1047Arg)	20.03
22	F	6	N	N	L-Hand: 5; R-Hand:1,2,3	*PIK3CA*	c.3140A > G (p.His1047Arg)	15.82
23	F	3	N	N	R-Hand: 5	*PIK3CA*	c.3140A > T (p.His1047Leu)	33.38
24	M	34	N	N	R-Hand: 1,2	*PIK3CA*	c.3140A > T (p.His1047Leu)	18.99

*M* male, *F* female, *Y* yes or present, *N* not present, *L* left, *R* right, *B* bilateral, *VAF* variant allele frequency, *NA* not available, *CCTN* cerebriform connective tissue nevi

Patients 1–4 had variable presentations of cerebriform connective tissue nevi adjacent to overgrown digits. Therefore, diagnoses of Proteus syndrome were established in these patients.

### Genetic characteristics

Through genetic testing and analysis, we achieved molecular diagnoses of all 24 patients. In the 4 patients who met the diagnostic criteria of Proteus syndrome, we identified an *AKT1* c.49G > A (p.Glu17Lys) variant, which is the only variant known to cause Proteus syndrome [12]. Variant allele frequencies (VAFs) of this *AKT1* mutation in affected tissues ranged from 10 to 22%, with an average variant frequency of 16%. No variant read was identified in blood DNA (Table 1).

In the 20 patients with isolated macrodactyly who did not meet the diagnostic criteria for Proteus syndrome, we identified and confirmed pathogenic variants in *PIK3CA* (Table 1). The most commonly observed variant was *PIK3CA* p.His1047Arg (N = 7), followed by p.Glu542Lys (N = 6), p.Glu545Lys (N = 2), p.His1047Leu (N = 2), p.Glu453Lys (N = 1), p.Gln546Lys (N = 1) and p.His1047Tyr (N = 1) (Table 1). VAFs in affected tissues ranged from 10 to 33% with an average of 21% (Table 1). None of these variants were identified in peripheral blood samples.

All seven variants have been previously reported to cause developmental disorders [10, 11, 13, 14], and either predicted or validated to have gain-of-function mechanisms. In vivo studies have demonstrated that mutations in *PIK3CA* are sufficient to induce oncogenic transformation in chicken embryo fibroblasts through enhancement lipid kinase activity and activation of mTOR and *AKT1* signaling [15]. All seven variants were absent from the Deciphering Disorders Involving Scoliosis and COmorbidities (DISCO, https://discostudy.org/) study composed of 4000 exome sequencing data of the Chinese population [16–18]. *PIK3CA* p.Glu453Lys, p.Glu542Lys, p.Gln546Lys and p.His1047Tyr were absent from the Genome Aggregation Database (gnomAD, https://gnomad.broadinstitute.org). *PIK3CA* p.Glu545Lys, p.His1047Leu, p.His1047Arg and *AKT1* p.Glu17Lys were present at extremely low frequencies in gnomAD, with an allele frequency of 4e-6. Despite previously reported observations of *PIK3CA* p.Gln546Lys, p.His1047Tyr and p.Glu453Lys mutations in other *PIK3CA*-related overgrowth syndromes (PROS) [11, 13, 19–21], our study represents the first time they are identified in isolated macrodactyly.

**Fig. 2:**
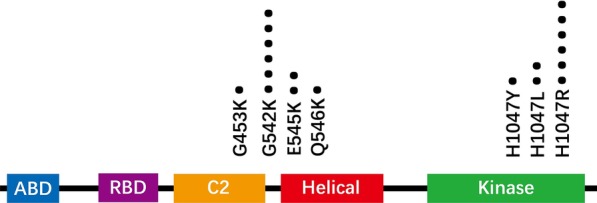
Distribution of *PIK3CA* variants identified in this study by functional domains. ABD: PI3K-ABD domain. RBD: PI3K-RBD domain. C2: C2 PI3K-type domain. Helical: PIK helical domain. Kinase: PI3K/PI4K kinase domain. A dot represents a *PIK3CA* variant identified in one patient

The PI3K protein has five functional domains, including PI3K-ABD, PI3K-RBD, C2 PI3K-type, PIK helical and PI3K/PI4K kinase domain. The p.Glu453Lys mutation is located in the C2 domain. Meanwhile, the p.Glu542Lys, p.Glu545Lys and p.Gln546Lys mutations occurr in adjacent amino acids of the helical domain. Lastly, the p.His1047Arg, p.His1047Tyr and p.His1047Leu mutations are located at the kinase domain of PIK3CA (Fig. 2). The majority of established functional variants of the *PIK3CA* cluster were found in the kinase and helical domains [22], which is consistent with our findings. In the 20 patients carrying pathogenic *PIK3CA* variants, 9 had variants of the helical domain and 10 had patients affecting the kinase domain, and only one patient had a variant affecting the C2 domain.

### Genotype–phenotype correlation

We then analyzed the potential correlation between subjects’ phenotypes (i.e. macrodactyly of the upper or the lower limb, the number of affected digits, with or without syndactyly) and the domain location of variant mutations in patients carrying the *PIK3CA* variant (Table 2).

**Table 2:** Genotype–phenotype correlation

	In helical domain	Not in helical domain
Affected limb		
Upper limb or both	0	7
Lower limb	9	4
* P*-value	0.005	
Affected digits		
< 3	5	8
≥ 3	4	3
* P*-value	0.642	

All 9 (100%) patients carrying variants in the helical domain of PI3K presented with macrodactyly in the lower limbs. In contrast, only 4 of 13 (36%) patients carrying variants outside of the helical domain (in the C2 domain or kinase domain) had a lower limb affected. These data demonstrates that helical domain variants are enriched in patients with lower limb overgrowth relative to all other variants (*P* = 0.005; determined by Fisher’s exact test). We also compared the VAFs between the two phenotypically different groups, but no significant correlation was observed.

13 patients had one or two affected digits, and five (38%) of these patients carried *PIK3CA* mutations located in the helical domain. 7 patients had more than two affected digits, 4 (57%) of whom had a *PIK3CA* mutation in the helical domain. While it may appear at first glance that patients with less than three affected digits were more likely to have a variant not located in the helical domain, and vice versa*,* this observation did not prove to be statistically significant (*P* = 0.642; determined by Fisher’s exact test).

## Discussion

In this study, 24 patients with macrodactyly underwent a targeted NGS-based sequencing and were identified to harbor mosaicism of either a pathogenic *PIK3CA* or *AKT1* variant. No pathogenic or likely-pathogenic variant was detected in other genes currently known to be associated with macrodactyly, implicating somatic mosaicism of *PIK3CA* or *AKT1* mutations as a predominant cause of isolated macrodactyly. This inference is supported by a separate recent study in which 9 out of 12 subjects with non-syndromic macrodactyly were positive for somatic mosaicism in *PIK3CA* [19].

Previous studies on the genetic causes of macrodactyly have largely applied Sanger sequencing toward mosaic variant detection [12, 23], which is not sensitive enough to detect low-level mosaicism [23]. Indeed, the mosaicism in our study patients no. 2, 3, 5, 8 and 20 would have been unlikely to be identified through this method (Additional file 1). In this report, we demonstrate that an NGS-based method can detect mosaicism even as low as 10%. Due to the limitations of Sanger sequencing in identifying mosaic variants and the wide spectrum of pathogenic variants (eight distinct variants identified in this study) [24], we employed NGS-based deep sequencing as our first-line in the molecular diagnosis of isolated macrodactyly.

*PIK3CA* mutations have been associated with a wide group of overgrowth disorders, with overlapping clinical manifestations. Macrodactyly has been reported in patients with CLAPO syndrome [8], CLOVES syndrome [4], KTS [25], as well as some forms of isolated lymphatic malformation or muscular hypertrophy [26]. CLAPO syndrome is characterized by capillary malformation of the lower lip, lymphatic malformation of the face and neck, and asymmetric overgrowth. In contrast, CLOVES syndrome presents with congenital lipomatous overgrowth, vascular malformations, epidermal nevi, and skeletal/scoliosis and spinal abnormalities. KTS is characterized by a triad of capillary malformation, venous varicosities and limb hypertrophy [27]. To differentially diagnose these separate PROS, clinicians must carefully evaluate patient’s phenotypes, as proper diagnosis has important implications in clinical management and follow-up.

Consistent with our report, the most commonly observed *PIK3CA* somatic variants in *PIK3CA*-related overgrowth syndromes (PROS) are p.His1047Arg, p.His1047Leu, p.Glu545Lys and p.Glu542Lys [13]. There are remarkable discrepancies between measured mosaic levels of samples collected from the same patient depending on whether Sanger sequencing or NGS is used [13, 26]. Seven variants of *PIK3CA* and one variant of *AKT1* that were identified in this study were previously described [13, 28]. However, we observed a significant heterogeneity in phenotypic presentations even for the same variant, which might be a consequence of the different times during development at which mutations were acquired [29]. The exact sites of variant-induced affliction in the human body might also play a role. It has been observed that PROS not involving the brain are usually caused by *PIK3CA* cancer hot-spot variants, e.g. p.Glu542Lys, p.His1047Arg and p.Glu453Lys [11, 13], while PROS involving the brain are usually caused by relatively rare variants. This phenomenon is also described in our report. Furthermore, we showed that macrodactyly in the upper limbs is caused primarily by variants outside the helical domain, whereas PROS involving the lower limbs are often a result of variants in the helical domain, and this association shows a robust statistical significance (*P* = 0.005). To evaluate the reproducibility of this result, we searched previous publications and found 13 patients from two studies with sufficient information to analyze genotype–phenotype correlation [14, 19]. While this sample size was too small to return a significant association (*P* = 0.497), the trend remains that all patients with mutations in the helical domain have only lower limbs affected. Indeed, when our data is combined with this previous data, the correlation is statistically significant (*P* = 0.002). Nevertheless, replication of this result in an independent cohort is still needed.

We found that VAFs of *PIK3CA* and *AKT1* in affected tissues were around 20%, and no variant was identified in blood samples, further supporting previous findings which suggests that *PIK3CA* mutations are generally undetectable in the blood of patients with Proteus syndrome or with PROS, excluding MCAP [12, 23, 30].

In conclusion, our findings demonstrate that isolated macrodactyly is predominantly caused by mosaic variants in *PIK3CA* or *AKT1*. We also show that patients with macrodactyly deformities of the upper limbs tend to carry *PIK3CA* variants with mutations outside the helical domain, while patients with deformities in the lower limbs have *PIK3CA* variants with mutations in the helical domain. With the advent of targeted therapy against the PI3K-*AKT1*-mTOR pathway, a deeper understanding of the pathogenesis of isolated macrodactyly holds great potential to benefit its effective management.

## Conclusions

This study reports the largest series of patients so far with *PIK3CA* /*AKT1*-associated macrodactyly, which includes 24 patients with isolated macrodactyly, all of whom carry mosaic *PIK3CA* or *AKT1* variants. Our findings expand the understanding of the mutational architecture and identifies novel genotype–phenotype correlations, providing insight into the genetic etiology of mosaic overgrowth syndromes and lends hope to improving precise management of these sets of syndromes.

## Methods

### Patient selection

Our study recruited 24 subjects clinically presenting with isolated macrodactyly, who were admitted to Jishuitan Hospital in 2018. Our criteria was limited to patients whose overgrowth did not exceed the limbs. Four subjects met the diagnostic criteria for Proteus syndrome and presented with skin findings and overgrowth limited to the hand or foot. The limb anomalies of these patients were evaluated through physical examinations and x-ray by experienced hand surgeons (WT, LS and YG).

### Tissue sampling and preparation

Abnormal adipose, skin and nerve tissues were collected during surgery. Genomic DNA was extracted from blood and collected tissues using the Dneasy Blood and Tissue Kit (QIAGEN, Germany) according to the manufacturer’s protocol.

### Genetic test and variant interpretation

A deep-targeted NGS protocol was performed on DNA extracted from blood and from surgically-removed abnormal tissues of all 24 subjects. Illumina paired-end libraries were prepared from DNA samples and were subjected to a customized NGS panel to detect somatic mutations on the mTOR-pathway-related genes, including *AKT1*, *AKT2*, *AKT3*, *PIK3CA* and *MTOR*, on the Illumina HiSeq X Ten platform (Illumina, US). The mean coverage for these mTOR-pathway-related genes was 1000 reads with more than 98% regions exceeding 100 reads. In-house developed Peking Union Medical College Hospital Pipeline (PUMP) and variant interpretation were performed following previously described methods [16–18].

All variants presumed to be pathogenic were subjected to Sanger sequencing. Variant-encoding amplicons were amplified by PCR from genomic DNA obtained from subjects, purified using an Axygen AP-GX-50 kit (lot no. 05915KE1) and sequenced by Sanger sequencing on an ABI3730XL instrument.

### Genotype–phenotype correlation analysis

Patients carrying variants in *PIK3CA* gene were selected to analyze potential genotype–phenotype correlation. We divided the patients into two groups based on the respective domain of an individual’s variant. We then compared the location of affected sites (at the upper or lower limbs) and the number of affected digits between the two groups.

SPSS Statistics V15.0 software was used for statistical analyses, and a p-value lower than 0.05 was considered statistically significant. Genotype–phenotype correlation was assessed using Fisher’s exact test and Student’s t test.

## Supplementary information

**Additional file 1.** Results of Sanger sequencing of the variants in *AKT1* and *PIK3CA*.

## Data Availability

The datasets used and analyzed during the current study are available from the corresponding author on reasonable request.

## Abbreviations

PIK3CA: Phosphatidylinositol 3-kinase catalytic subunit alpha
AKT1: AKT serine/threonine kinase 1
PI3K: Phosphatidylinositol 3-kinase
NGS: Next generation sequencing
VAFs: Variant allele frequencies
PROS: *PIK3CA*-related overgrowth syndrome
MCAP: Megalencephaly-capillary malformation
