# Supplementary material for: Phenotypic and genetic spectrum of isolated macrodactyly: somatic mosaicism of PIK3CA and AKT1 oncogenic variants
Source: Orphanet J Rare Dis. 2020 Oct 14;15:288. doi: 10.1186/s13023-020-01572-9 (PMC7556951; doi:10.1186/s13023-020-01572-9)

Patient 1

*AKT1*1: c.49G>A(p.Glu17Lys)

VAF (identified through NGS)=22.03%


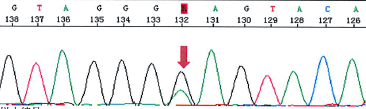


Patient 2

*AKT1*1: c.49G>A(p.Glu17Lys)

VAF (identified through NGS)=11.16%


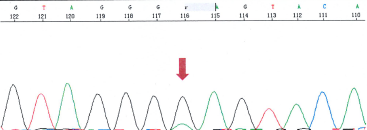


Patient 3

*AKT1*1: c.49G>A(p.Glu17Lys)

VAF (identified through NGS)=9.93%


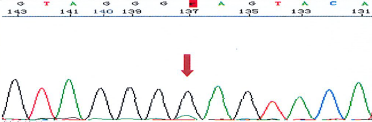


Patient 4

*AKT1*1: c.49G>A(p.Glu17Lys)

VAF (identified through NGS)=20.57%


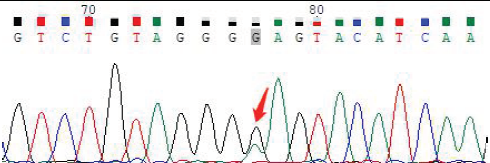


Patient 5

*PIK3CA*: c.1357G>A(p.Glu453Lys)

VAF (identified through NGS)=11.10%


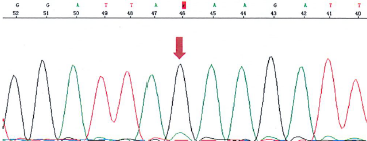


Patient 6

*PIK3CA*: c.1624G>A(p.Glu542Lys)

VAF (identified through NGS)= 24.48%


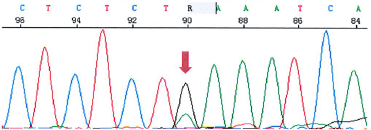


Patient 7

*PIK3CA*: c.1624G>A(p.Glu542Lys)

VAF (identified through NGS)= 17.15%


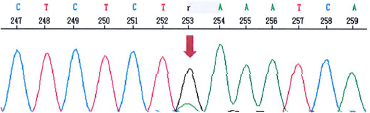


Patient 8

*PIK3CA*: c.1624G>A(p.Glu542Lys)

VAF (identified through NGS)= 20.95%


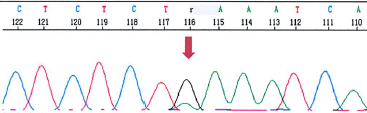


Patient 9

*PIK3CA*: c.1624G>A(p.Glu542Lys)

VAF (identified through NGS)= 25.63%


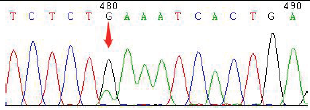


Patient 10

*PIK3CA*: c.1624G>A(p.Glu542Lys)

VAF (identified through NGS)= 17.10%


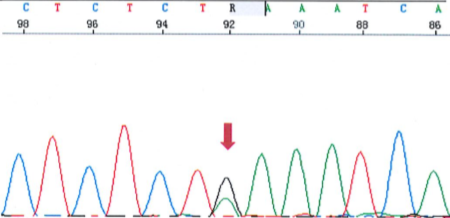


Patient 11

*PIK3CA*: c.1624G>A(p.Glu542Lys)

VAF (identified through NGS)= 27.58%


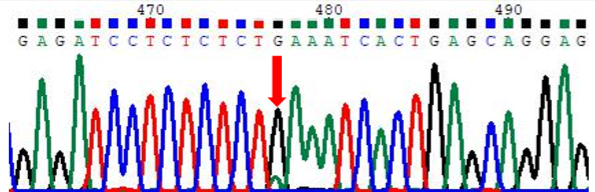


Patient 12

*PIK3CA*: c.1633G>A(p.Glu545Lys)

VAF (identified through NGS)= 19.11%


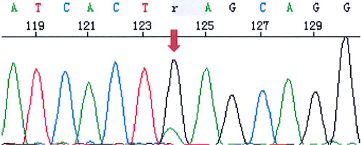


Patient 13

*PIK3CA*: c.1633G>A(p.Glu545Lys)

VAF (identified through NGS)= 27.31%


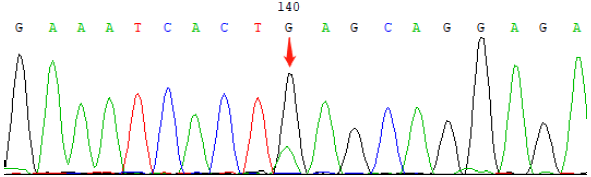


Patient 14

*PIK3CA*: c.1636C>A(p.Gln546Lys)

VAF (identified through NGS)= 24.50%


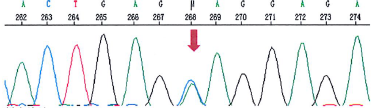


Patient 15

*PIK3CA*: c.3139C>T(p.His1047Tyr)

VAF (identified through NGS)= 18.94%


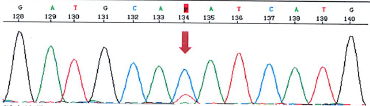


Patient 16

*PIK3CA*: c.3140A>G(p.His1047Arg)

VAF (identified through NGS)= 23.29%


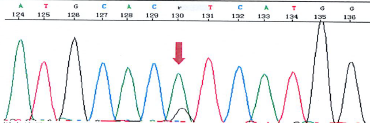


Patient 17

*PIK3CA*: c.3140A>G(p.His1047Arg)

VAF (identified through NGS)= 17.79%


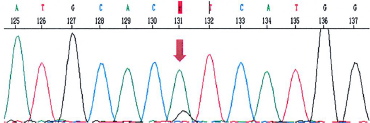


Patient 18

*PIK3CA*: c.3140A>G(p.His1047Arg)

VAF (identified through NGS)= 21.45%


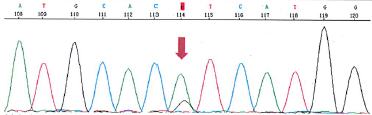


Patient 19

*PIK3CA*: c.3140A>G(p.His1047Arg)

VAF (identified through NGS)= 25.57%


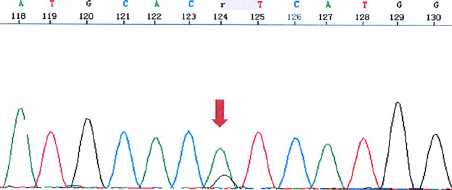


Patient 20

*PIK3CA*: c.3140A>G(p.His1047Arg)

VAF (identified through NGS)= 10.36%


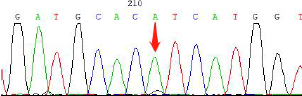


Patient 21

*PIK3CA*: c.3140A>G(p.His1047Arg)

VAF (identified through NGS)= 20.03%


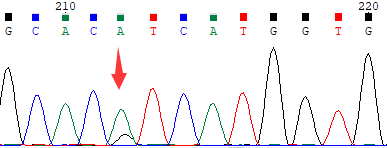


Patient 22

*PIK3CA*: c.3140A>G (p.His1047Arg)

VAF (identified through NGS)=15.82%


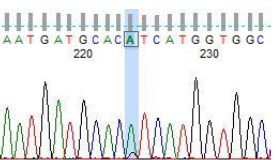


Patient 23

*PIK3CA*: c.3140A>G(p.His1047Arg)

VAF (identified through NGS)= 33.38%


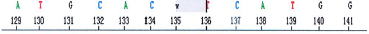


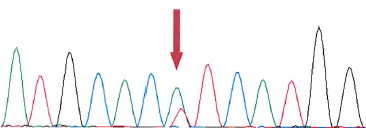


Patient 24

*PIK3CA*: c.3140A>G(p.His1047Arg)

VAF (identified through NGS)= 18.99%


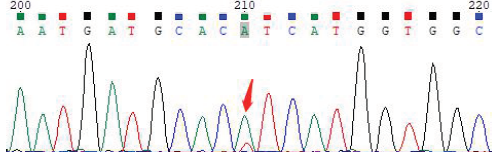

Supplement: Supplementary file 1 — Additional file 1. Results of Sanger sequencing of the variants in AKT1 and PIK3CA. [file 13023_2020_1572_MOESM1_ESM.docx]
